# Antioxidant modulation of nevirapine induced hepatotoxicity in rats

**DOI:** 10.1515/intox-2015-0002

**Published:** 2015-03

**Authors:** Olufunsho Awodele, Temidayo Popoola, Kunle Rotimi, Victor Ikumawoyi, Wahab Okunowo

**Affiliations:** 1 Department of Pharmacology, Therapeutics and Toxicology, College of Medicine, University of Lagos, PMB 12003, Lagos-Nigeria; 2 Department of Biochemistry, College of Medicine University of Lagos, PMB 12003, Lagos-Nigeria

**Keywords:** ART, nevirapine, jobelyn, vitamin C, vitamin E

## Abstract

HIV/AIDS related mortality has been dramatically reduced by the advent of antiretroviral therapy (ART). However, ART presents with associated adverse effects. One of such adverse effects is hepatotoxicity observed with nevirapine (NVP) containing ART. Since previous studies showed that NVP hepatotoxicity may be due to oxidative stress via generation of oxidative radicals, this study sought to evaluate the protective effects of antioxidants in alleviating NVP induced hepatotoxicity. Rats were divided into 6 groups with 8 animals per group and received doses of the antioxidants jobelyn (10.7 mg/kg/day), vitamin C (8 mg/kg/day), vitamin E (5 mg/kg/day) and/or NVP (6 mg/kg/day) for 60 days. The animals were sacrificed on day 61 by cervical dislocation, blood samples were collected for biochemical and hematological examination. The liver of the sacrificed animals was weighed and subjected to histopathological examination. There was a statistically significant (*p*<0.05) elevation in MDA level observed in the NVP group as compared with control. The results further showed non-significant decreases in the levels of MDA in the NVP plus antioxidant groups, except vitamin C, when compared with the NVP alone group. Vitamin E and Vitamin E plus C treated groups showed significantly (*p*<0.05) higher levels of SOD, CAT and GSH. The results also showed statistically significantly (*p*<0.05) lower levels of ALT and AST in the antioxidant treated groups There was an observed significantly (*p*<0.05) higher level of TP and urea in the antioxidant treated rats. A significantly (*p*<0.05) higher white blood cell count was observed in the antioxidant groups. Histopathological assessment of the liver extracted from the rats showed no visible pathology across the groups. Observations from this study suggest a potentially positive modulatory effect of antioxidants and may be indicative for the inclusion of antioxidants in nevirapine containing ART.

## Introduction

The human immunodeficiency virus (HIV) is a retrovirus known to be the cause of acquired immune deficiency syndrome (AIDS). About 39 million people globally are living with HIV with high morbidity/mortality data in sub-Saharan Africa including Nigeria (WHO 2006). HIV/AIDS related mortality has however been dramatically reduced by the advent of antiretroviral therapy (ART) (Emejulu *et al*., 2010). Current ART guidelines involve the combination of antiretroviral drugs – referred to as highly active antiretroviral therapy (HAART) – which hamper the growth of the HIV virus, eventually leading to decreased viral load, thereby prolonging the life span and improving the quality of life of the patient (Hughes *et al*., 2011).

A drawback to the use of ART includes adverse effects associated with these antiretroviral drugs (Elias *et al*., 2012). One of such adverse effects is hepatotoxicity, frequently reported in patients taking nevirapine (NVP) containing ART (WHO, 2006). Case reports, clinical trials and other studies have linked NVP with hepatotoxicity in HIV patients taking NVP containing ART (Elias *et al*., 2013). NVP has been reported to be associated with early hypersensitivity reactions and late onset hepatotoxicity leading to elevation of liver enzymes (Soriano *et al*., 2008; Elias & Brambaifa, 2013; Elias *et al*., 2013).

The WHO therefore recommends the use of NVP with caution and regular monitoring in patients who have baseline elevations of liver enzymes and in co-infections with hepatitis B or C viruses (WHO, 2006). In January and March 2005, the US Food and Drug Administration (USFDA, 2005) and the European Medicines Agency (EMEA, 2005), respectively, issued warnings in NVP package inserts against the initiation of NVP in adult women with CD4 count above 250 cells/ml or in men with a count of above 400 cells/ml, because of a higher risk of hepatotoxicity.

Oxidative stress (defined as an imbalance between the antioxidant and pro-oxidant systems, with the shift towards the pro-oxidant system) is a common feature in liver hepatotoxicity (Elias *et al*., 2013). The term is usually used to describe the damage caused by reactive oxygen species (ROS) to tissues or organs. Collectively, ROS can lead to oxidation of proteins, DNA, peroxidation of lipids and ultimately cell death. Evidence shows oxidative stress as a factor in the progression of drug induced hepatotoxicity (Yamamoto *et al*., 2005; Kashou and Agarwal, 2011). Recent studies have also shown that antiretroviral drugs (including NVP) induce oxidative stress via generation of oxidative radicals, which may be associated with their toxicological effects (Valle *et al*., 2013). Given that the antioxidant system prevents tissue damage (and consequent loss of function) caused by oxidative radicals during a period of persistent oxidative stress, and that the provision of simple, inexpensive micronutrient supplements as an adjunct to HAART may have several cellular and clinical benefits, such as reduction in mitochondrial toxicity and oxidative stress (Drain *et al*., 2007), it may be important to evaluate the role of this system (including exogenous antioxidants) in ameliorating drug (NVP) induced hepatotoxicity.

This study seeks to evaluate the protective antioxidative effects of different exogenous antioxidants (jobelyn, vitamin C and vitamin E) in alleviating NVP induced hepatotoxicity. The investigation into possible tissue protective roles of these antioxidants was done by measurement of oxidative stress parameters, serum levels of liver function enzymes and by evaluating the histopathological features of the liver in the experimental animals used for the study.

The findings from this study are expected to provide a scientific basis for further investigations into the inclusion of exogenous antioxidants in NVP containing regimens currently used in antiretroviral therapy.

## Methodology

### Drugs

Nevirapine was obtained from the HIV/AIDS Clinic of the University of Lagos Teaching Hospital, while vitamin E, vitamin C and jobelyn *(Sorghum bicolor* leaf extract) were obtained from Health – Plus Pharmacy, Domino, Yaba.

### Animals

The animals used in this study were 4–6 week-old male albino rats with average weight of 140 g. They were obtained from the Laboratory Animal Centre of the College of Medicine, University of Lagos, Idi-Araba. They were housed and kept in standard environmental conditions with access to standard rodent feed and clean water (pH 7.0) ad libitum and acclimatized for a period of two weeks before experimental procedures. The investigation conforms to The Guide for the Care and Use of Laboratory

Animals published by the U. S. National Institutes of Health (NIH Publication No. 85-23, revised 1996) for studies involving experimental animals.

### Treatment groups

The animals were divided into six groups, each containing eight rats. The antioxidant groups were pretreated with jobelyn (10.7 mg/kg), vitamin C (8 mg/kg) or vitamin E (5 mg/kg) for two weeks before the administration of nevirapine (6 mg/kg). All treatments were administered orally.

- *Group 1* (control): normal saline (10 ml/kg/day) throughout the treatment period
- *Group 2:* nevirapine (6 mg/kg/day) for a period of 60 days
- *Group 3:* jobelyn (10.7 mg/kg/day) for two weeks then a combination of nevirapine (6 mg/kg /day) and jobelyn (10.7 mg/ kg/day) for 60 days
- *Group 4:* vitamin C (8 mg/kg/day) for two weeks then a combination of nevirapine (6 mg/kg/day) and vitamin C (8 mg/kg/day) for 60 days
- *Group 5:* vitamin E (5 mg/kg/day) for two weeks then a combination of nevirapine (6 mg/kg/day) and vitamin E (5 mg/kg/day) for 60 days
- *Group 6:* vitamin C (8 mg/kg/day) and vitamin E (5 mg/kg/day) for two weeks followed by a combination of nevirapine (6 mg/kg/day), vitamin C (8 mg/kg/day) and vitamin E (5 mg/kg/day) for 60 days

### Biochemical and hematological examination

On the 61^st^ day after termination of administration of the drugs, the rats were anesthetized and sacrificed by cervical dislocation. Blood samples were collected through the retro-orbital plexus vein of the eye for biochemical and hematological determination.

The fully automated clinical chemistry analyzer (Hitachi 912, Boehringer Mannheim, Germany) was used to determine the levels of aspartate aminotransferase (AST), alanine aminotransferase (ALT), alkaline phosphatase (ALP), urea, creatinine, albumin, total protein, bilirubin, cholesterol, triglyceride, serum catalase (CAT), superoxide dismutase (SOD), reduced glutathione (GSH), malondialdehyde (MDA) and the fully automated clinical hematological analyzer (Pentra-XL 80, Horiba ABX, USA) was used to determine the levels of white blood cells, red blood cells, hemoglobin, hematocrit (packed cell volume), platelet, mean cell hemoglobin concentration (MCHC) and mean cell hemoglobin (MCH).

### Histopathological examination

Qualitative data on liver weight of the albino rats were assessed by carefully dissecting the liver from the sacrificed animal into normal saline contained in a sample bottle. Isolated livers were dried and weighed using Mettler sensitive weighing balance. The weight of each liver was standardized to 100 g body weight of each animal.

After weighing, each liver was fixed in 10% formalin in labeled bottles. Tissues were processed routinely and embedded in paraffin wax. Sections of 5 μ thickness were cut, stained with hematoxylin and eosin and examined under the light microscope by a pathologist.

### Statistical analysis

Results were expressed as mean ± SEM. The data were subjected to one way analysis of variance (ANOVA) test and differences between samples were determined by Dennett's Multiple Comparison Test, using the Graph Pad Prism (statistical) soft ware. Results were considered to be significant at *p*<0.05.

## RESULTS

Table 1 presents the mean weight (g) ± SEM of the liver extracted from male rats following administration of the drugs. The results show no statistically significant (*p*<0.05) variation in liver weight among the treatment groups.

**Table 1:** Effect of nevirapine and exogenous antioxidants on liver weight.

Treatment	Weight (g)/100 g BW
Control	7.21±1.148
NVP	6.61±0.822
Jobelyn + NVP	6.23±1.007
Vitamin C + NVP	5.91±1.616
Vitamin E + NVP	7.33±1.337
Vitamin C + Vitamin E + NVP	7.29±1.748

Table 2 shows the effects of drug treatments on liver antioxidant enzymes and MDA. There was a statistically significantly (*p*<0.05) higher level of MDA in the NVP group as compared with the control group. The combination of NVP and antioxidant groups showed non-significant decreases in MDA levels as compared with NVP alone, except the vitamin C combination group. The vitamin E treated group showed a statistically significantly (*p*<0.05) higher level of SOD, CAT and GSH when compared with other groups, with the exception of the vitamin C plus vitamin E treated group for SOD. The vitamin C plus vitamin E treated group also showed statistically significant higher levels of GSH and SOD when compared with the control. There were no statistically significant variations (*p*<0.05) in liver weight among the groups.

**Table 2:** Effect of exogenous antioxidants on in vivo antioxidant enzymes and MDA levels in liver samples of treated rats.

Treatment	CAT(U/mg)	SOD(U/mg)	GSH(U/mg)	MDA(U/mg)
Control	13.02±0.505	1.68±0.0423	0.39±0.013	0.0458±0.002
NVP	14.25±2.686	1.926±2.686	0.52±0.078	0.1602±0.020^a^
Jobelyn + NVP	11.57±1.583	2.418±0.151	0.56±0.041	0.1362±0.022^a^
Vitamin C + NVP	12.67±0.708	2.30±0.160	0.51±0.039	0.1758±0.005^a^
Vitamin E+ NVP	23.63±0.742^a,b,c,d^	3.67±0.262^a,b,c,d,f^	0.81±0.070^a,b,c,d^	0.1446±0.009^a^
Vitamin E + Vitamin C + NVP	17.16±3.730	2.51±1.669^a^	0.66±0.050^a^	0.1472±0.012^a^

Data are expressed as mean ± SEM (n=8)

a represents results where *p*<0.05 as compared with control,

b represents results where *p*<0.05 as compared with NVP

c represents results where *p*<0.05 compared with jobelyn + NVP

d represents results where *p*<0.05 as compared with vitamin C + NVP

e represents results where *p*<0.05 as compared with vitamin E + NVP

f represents results where *p*<0.05 as compared with vitamin C + vitamin E + NVP

**NVP** is nevirapine; **CAT** is serum catalase; **SOD** is superoxide dismutase; **GSH** is reduced glutathione; **MDA** is malondialdehyde

SEM is standard error of mean

Table 3 shows the effects of drug treatment on serum biochemical parameters. There were statistically significantly (*p*<0.05) lower levels of ALT, AST in the antioxidant treated groups and the control group compared with the nevirapine group, with an exception observed in the jobelyn group where there was no significantly lower level of AST and ALT when compared with the NVP group; however all the antioxidant groups show statistically significantly (*p*<0.05) higher levels of AST and ALT when compared with the control group. There was also a significantly (*p*<0.05) higher level of AST in the jobelyn group when compared with the vitamin C and vitamin E groups. A significantly (*p*<0.05) higher level of TP and urea was recorded in the antioxidant treated rats and control group compared with the NVP group.

**Table 3a:** Effect of exogenous antioxidant on serum biochemical parameters of treated rats.

Treatment	ALT (μ/l)	AST (μ/l)	ALP (μmol/l)	BIL (μmol/l)	CHOL (mmol/l)	TG (mmol/l)
Control	26.76±6.418^b^	112.16±15.740^b^	153.20±33.214^d^	18.44±0.262	2.49±0.152	1.22±0.048
NVP	56.10±1.661	213.82±4.166	218.96±13.413	43.20±24.251	2.04±0.389	1.89±0.983
Jobelyn + NVP	39.62±0.7977^b^	190.66±2.638^a^	188.12±15.235	20.28±1.192	2.28±0.162	2.16±0.764
Vitamin C + NVP	37.10±3.713^b^	139.54±2.314^a,b,c^	203.24±12.902	17.66±5.425	2.25±0.143	2.13±1.027
Vitamin E + NVP	40.50±1.151^b^	153.58±2.487^a,b,c^	274.12±22.208	16.64±2.104	2.30±0.114	2.08±0.777
Vitamin E + Vitamin C + NVP	39.70±3.041	168.73±2.963^a,b^	176.67±33.031	17.30±1.201	1.90±0.241	1.24±0.397

Data are expressed as mean ± SEM (n=8)

a represents results where *p*<0.05 as compared with control,

b represents results where *p*<0.05 as compared with NVP

c represents results where *p*<0.05 compared with jobelyn + NVP

d represents results where *p*<0.05 as compared with vitamin C + NVP

e represents results where *p*<0.05 as compared with vitamin E + NVP

f represents results where *p*<0.05 as compared with vitamin C + vitamin E + NVP

**NVP** is nevirapine; **AST** is aspartate transaminase; **ALT** is alanine transaminase; **BIL** is bilirubin; **ALP** is alkaline phosphatase; **CHOL** is cholesterol;

**TG** is triglycerides; SEM is standard error of mean

**Table 3b:** Effect of exogenous antioxidant on serum biochemical parameters of treated rats.

Treatment	ALB(g/l)	TP(g/l)	UREA(mmol/l)	CREA(μmol/l)	GLU(mmol/l)
Control	41.88±3.155	67.06±2.298	4.26±0.802^b^	43.67±3.833	1.48±0.657
NVP	38.60±13.892	54.54±23.346	2.82±1.195	26.57±8.503	2.20±1.155
Jobelyn + NVP	42.20±0.418	66.72±1.003^b^	5.08±0.719^b^	35.07±5.091	2.26±0.4278
Vitamin C + NVP	40.66±2.585	68.08±1.665^b^	4.64±1.174^b^	40.07±4.840	1.82±0.536
Vitamin E + NVP	35.96±4.899	65.22±2.632	4.18±0.259^b^	42.88±4.534	1.94±0.770
Vitamin E + Vitamin C + NVP	41.33±2.714	56.0±21.398	4.50±0.954^b^	35.13±3.477	1.93±0.3055

Data are expressed as mean ± SEM (n=8)

a represents results where *p*<0.05 as compared with control,

b represents results where *p*<0.05 as compared with NVP

c represents results where *p*<0.05 compared with jobelyn + NVP

d represents results where *p*<0.05 as compared with vitamin C + NVP

e represents results where *p*<0.05 as compared with vitamin E + NVP

f represents results where *p*<0.05 as compared with vitamin C + vitamin E + NVP

**NVP** is nevirapine; **ALB** is albumin; **TP** is total protein; **CREA** is creatinine; **GLU** is glucose; SEM is standard error of mean

Table 4 shows the *in vivo* effects of drug treatments on hematological parameters. There was a statistically significantly (*p*<0.05) lower level of white blood cells in the NVP and antioxidant groups compared with the control group. A significantly higher level of white blood cells was found in the antioxidant groups compared with the NVP group. Other hematological parameters show no statistically significant (*p*<0.05) variations among the groups.

**Table 4:** Effect of exogenous antioxidants on hematological parameters of treated rats.

Treatment	RBC (10^9^/l)	WBC (10^12^/l)	PCV (%)	PLAT (10^9^/l)	HB (g/dl)	MCH (Pg)	MCHC(g/dl)
Control	7.42±0.520	11.2±0.524	49.32±3.703	638.40±48.967	13.00±1.068	17.42±0.712	26.14±0.847
NVP	6.41±0.596	5.82±0.276^a,^	42.56±8.398	672.0±121.050	11.80±1.416	18.28±0.740	26.48±0.760
Jobelyn ± NVP	6.65±0.60	8.76±1.144^a,b^	50.0±2.957	685.0±40.503	13.22±0.545	18.02±0.853	26.10±0.718
Vitamin C ± NVP	7.41±0.763	9.06±0.992^a,b^	45.70±6.135	738.20±115.820	12.28±1.507	17.04±0.470	26.12±0.492
Vitamin E ± NVP	6.91±0.292	9.18±0.383^a,b^	47.78±1.103	712.40±110.790	12.66±0.654	17.72±1.089	25.50±2.001
Vitamin E ± Vitamin C ± NVP	7.53±0.957	8.36±0.971^a,b^	49.77±1.050	670.33±68.149	13.03±0.586	18.13±0.208	25.90±1.179

Data are expressed as mean ± SEM (n=8)

a represents results where *p*<0.05 as compared with control,

b represents results where *p*<0.05 as compared with NVP

c represents results where *p*<0.05 as compared with jobelyn + NVP

d represents results where *p*<0.05 as compared with vitamin C + NVP

e represents results where *p*<0.05 as compared with vitamin E + NVP

f represents results where *p*<0.05 as compared with vitamin C + vitamin E + NVP

**NVP** is nevirapine; **RBC** is red blood cells; **WBC** is white blood cells; **HB** is hemoglobin; **PCV** is packed cell volume; **PLAT** is platelets; **MCH** is mean corpuscular hemoglobin; **MCHC** is mean corpuscular hemoglobin concentration; **SEM** is standard error of mean.

Histopathological assessment of the liver extracted from the rats after drug exposure showed no visible pathology across the groups (Table 5).

**Table 5:** Summary of histopathological assessment of rat liver exposed to drug treatment.

Treatment	OBSERVATION
Control	Normal
NVP	Normal
Jobelyn ± NVP	Normal
Vitamin C ± NVP	Normal
Vitamin E ± NVP	Normal
Vitamin E ± Vitamin C ± NVP	Normal

## Discussion

Nevirapine is an important component of HAART and it has been clinically proven to be hepatotoxic in HIV patients taking NVP containing ART (Elias *et al*., 2013). Our previous study (Awodele *et al*., 2011) showed that the hepatotoxic effect of zidovudine plus combined anti-TB drugs, possibly due to free radical generation, was modulated by NEUTROSEC^®^ (a combination of vitamins and amino acids) in animal models. Due to the relevance and interesting results obtained from the above mentioned study, a clinical trial model of this research was proposed with the goal to improve pharmacotherapy and limit adverse effects (hepatic toxicity) of patients on these medications. The present study was designed to evaluate the possible modulation of jobelyn and other nutritional antioxidants in nevirapine (NVP) induced hepatotoxicity.

The results showed statistically significant (*p*<0.05) increase in MDA concentration in the NVP treated group compared with the control group. As the quantitation of MDA is widely used as an indicator of lipid peroxidation (Simsek *et al*., 2006), this finding indicates an NVP induced increase in lipid peroxidation and agrees with the suggestion of Zalen *et al*., 2010. Lipid peroxy radicals have been shown to increase cell membrane permeability, decrease cell membrane fluidity, inactivate membrane proteins and cause a loss of polarity across mitochondrial membranes, ultimately leading to mitochondrial toxicity, a mechanism attributable to NVP induced hepatotoxicity (Wei *et al*., 1998; Elias *et al*., 2013). There were however no statistically significant differences only slight reductions in MDA levels between the NVP group and the NVP plus antioxidant treated groups, suggesting that an increase in the concentrations of antioxidants may be needed to induce significant reductions.

GSH, a major endogenous antioxidant participates directly in neutralizing free radicals and ROS as well as maintaining antioxidants such as vitamin C and E in their reduced active form (Scholz *et al*., 1964). Since vitamin E inhibits glutathione S-transferase (GST) in humans (Haaften *et al* 2001), the high level of GSH in the vitamin E plus NVP group may be secondary to GST inhibition, thereby preventing the conjugation of GSH catalyzed by GST (Douglas, 1987; Costagliola & Menzione, 1990) leading to accumulation of GSH.

The high level of GSH will definitely contribute to the antioxidant system (Onyema *et al*., 2006). When vitamin E is depleted due to its oxidation, glutathione reduces tocopheroxyl radicals to tocopherol and is itself oxidized, however in the presence of exogenous supply of vitamin E, glutathione is maintained in its reduced state (Hess, 1993). This may also account for the significantly higher level of GSH in the vitamin E treated group compared with the NVP treated group, as obtained in the study.

The statistically significantly higher levels of the other enzymes, SOD and CAT, observed in the vitamin E plus NVP treated group when compared with the control indicate that vitamin E stimulates the antioxidant systems in the rat liver (Onyema *et al*., 2006; Zelen *et al*., 2010). Since higher levels of MDA in NVP exposed rats suggest that NVP stimulated lipid peroxidation, which will result in the formation of aldehydic and reactive by-products, and which, in turn, decrease the GSH content (Gurer *et al*., 2001), it seems plausible that vitamin E can improve the antioxidant defense system via inhibiting the lipid peroxidation process, thereby mitigating the consumption of GSH. The same mechanism, rather than a direct effect on the enzymes, could also explain the beneficial effects of vitamin E on CAT and SOD (Gurer *et al*., 2001).

Vitamin C supplementation has been reported to increase the level of GSH (Carol *et al*., 1993), however in our study the NVP plus vitamin C group showed a statistically nonsignificantly (*p*<0.05) higher level of GSH compared with the other groups. The vitamin C/vitamin E/NVP combination also showed no significant effects on CAT compared with other groups; there were however, statistically significantly higher levels of GSH and SOD compared with the control. Overall, these results suggest that a combination of vitamin C/vitamin E offers no advantage over vitamin E or even vitamin C alone in modulating oxidative stress associated NVP induced hepatotoxicity. Jobelyn showed no significant increase in the antioxidant systems when compared with the other exogenous antioxidants; this suggests that jobelyn has lower antioxidative effects in NVP induced hepatotoxicity when compared with vitamins E and C.

In an evaluation of the effect of micronutrient supplementation in HIV-positive persons receiving HAART, a non-randomized intervention study assessed the effects of either a low-dose or high-dose antioxidant regimen (mainly vitamins A, C, and E and selenium) for 12 weeks on antioxidant defenses, oxidative stress, and plasma viral load (Batterham *et al*., 2001). The results showed that antioxidant supplements significantly increased antioxidant defenses but had no significant effect on oxidative stress or plasma viral load. No significant differences were observed between rats supplemented with low-dose and those supplemented with high-dose antioxidants. A summary of the review work by Drain *et al*. (2007) suggests that intervention studies with antioxidants found increased oxidative defenses, but only one of these studies found decreased oxidative stress.

However, Drain *et al*. (2007) pointed out that micronutrients may play a role in reducing mitochondrial dysfunction and metabolic complications, which are commonly experienced by HIV-positive persons receiving HAART. This conclusion was based on reports which state that selenium supplements were shown to stimulate glutathione peroxidase activity (a measure of antioxidant defenses) and reduce NF-κB activation in HIV-1 infected cells (Sappey *et al*., 1994; Taylor *et al*., 1997; Zhao *et al*., 2000), along with a study of 120 HIV-positive adults receiving HAART that found that a greater total intake of vitamin E was associated with fewer outcomes of HAART associated metabolic complications (including body fat redistribution, dyslipidemia, and insulin resistance) due to changes in the ratio of plasma reduced to oxidized glutathione and oxygen free radicals (Gavrila *et al*., 2003).

Serum markers of hepatocellular injury ALT, AST and ALP in the NVP group were significantly (*p*<0.05) higher when compared with the control group and the antioxidant groups. The high levels of these markers observed in the NVP group might be a result of hepatic injury caused by NVP induced oxidative stress (Elias *et al*., 2013). The lower level of AST in the Vitamin E and Vitamin C treated groups was statistically significant when compared with control, NVP and jobelyn groups. These findings further suggest that jobelyn offers no advantage in modulating NVP induced hepatotoxicity compared with vitamin C and vitamin E. The Vitamin C/vitamin E combination as antioxidant shows no advantage over vitamin C and vitamin E used individually in preventing nevirapine induced elevation of AST.

It should however be noted that ALT, AST and ALP levels are usually elevated also in cases of injury to other organs, like kidney, brain, and heart (Bain, 2003). The value of total proteins is helpful in differentiating between hepatic injury and injury to other organs as the majority of plasma proteins are produced in the liver (Thapa & Walia, 2007). There was a significantly (*p*<0.05) lower level of total protein in the NVP group when compared with the vitamin C plus NVP and the jobelyn plus NVP groups, indicative of hepatocellular injury due to NVP, since the total protein level is often reduced during hepatocellular injury (Singh *et al*., 2011).

There was also a significantly (*p*<0.05) lower level of urea in the NVP group compared with the antioxidant group, suggesting that the exogenous antioxidants (vitamin E, vitamin C and jobelyn) protected against NVP induced decreased urea production. In hepatotoxicity there is generally a lower level of urea (Singh *et al*., 2011).

Hematological analysis showed slight variations among the groups which were not statistically significant, with the exception of the white blood cell count which showed a statistically significant (*p*<0.05) lower count for the NVP group compared to other groups, suggesting that NVP induces a low white blood cell count. This is at variance with the work of Umar *et al*. (2007) where nevirapine was found to have no effect on the white blood cell count.

Histological examination revealed no visible pathology in any of the groups studied. This may be due to a homeostatic mechanism through which restoration takes place after tissue injury (Clancy *et al*., 2002) or the tissue suffered only minor injury due to short exposure to NVP. Biochemical alterations and anomalies have been repeatedly reported to precede marked organ damage after long-term continuous exposure.

## Conclusion

Nevirapine remains an essential component in antiretroviral therapy despite evidence that it carries a risk of hepatotoxicity associated with oxidative stress. Observations from the presented study suggest potential positive modulatory effects of antioxidants and may be indicative for the inclusion of antioxidants, particularly vitamin E, in nevirapine containing ART. However, further studies are needed to ascertain these findings.
